# Arginine side chain interactions and the role of arginine as a gating charge carrier in voltage sensitive ion channels

**DOI:** 10.1038/srep21759

**Published:** 2016-02-22

**Authors:** Craig T. Armstrong, Philip E. Mason, J. L. Ross Anderson, Christopher E. Dempsey

**Affiliations:** 1 School of Biochemistry, Bristol University, Bristol BS8 1TD, UK; 2 Institute of Organic Chemistry and Biochemistry, Academy of Sciences of the Czech Republic and Center for Biomolecules and Complex Molecular Systems, 16610 Prague 6, Czech Republic

## Abstract

Gating charges in voltage-sensing domains (VSD) of voltage-sensitive ion channels and enzymes are carried on arginine side chains rather than lysine. This arginine preference may result from the unique hydration properties of the side chain guanidinium group which facilitates its movement through a hydrophobic plug that seals the center of the VSD, as suggested by molecular dynamics simulations. To test for side chain interactions implicit in this model we inspected interactions of the side chains of arginine and lysine with each of the 19 non-glycine amino acids in proteins in the protein data bank. The arginine guanidinium interacts with non-polar aromatic and aliphatic side chains above and below the guanidinium plane while hydrogen bonding with polar side chains is restricted to in-plane positions. In contrast, non-polar side chains interact largely with the aliphatic part of the lysine side chain. The hydration properties of arginine and lysine are strongly reflected in their respective interactions with non-polar and polar side chains as observed in protein structures and in molecular dynamics simulations, and likely underlie the preference for arginine as a mobile charge carrier in VSD.

Lysine and arginine are the two positively charged amino acids in proteins that have high aqueous pKa’s (~10.5 for Lys^1^ and ~13.8 for Arg^2^) indicating a strong propensity to carry charge at physiological pH. These amino acids have profound roles in protein structure and function that involve electrostatic interactions and protein solvation. The very high pKa of Arg results from delocalization of the positive charge within the π-bonded system of the side chain guanidinium ion; so stable is the guanidinium that the Arg side chain remains protonated when partially buried within protein structures or membranes^3^,^4^. This contrasts with Lys where the charge is largely focused on the terminal aliphatic amino group and the side chain is readily deprotonated in proteins^1^.

The physicochemical properties of these side chains underlie the roles of Arg and Lys in diverse biological structures and functions. This is particularly marked for the voltage-sensor domains (VSD) of voltage sensitive ion channels and enzymes that contain a mobile transmembrane helical segment (S4) which moves up and down through the membrane plane as the VSD responds to changes in membrane potential^5^,^6^. The S4 helix contains a repeating triplet motif consisting of positively charged amino acids (Lys or Arg) separated by two hydrophobic amino acids; it is the interaction of S4 positive charges with the membrane potential that drives the conformational changes underlying membrane voltage sensitivity. In voltage-mediated transitions the positive side chains pass through a hydrophobic “plug”^7^ or “gasket”^8^ which separates the internal and external regions of the VS and provides a barrier to passage of ions, focusing the membrane potential across a narrow region of the membrane. The mobile charges on S4 that pass through the hydrophobic plug (gating charges) are almost exclusively carried on arginine side chains; indeed it is possible that lysine is never a carrier of mobile charges across the hydrophobic plug in membrane voltage sensors.

The molecular basis for the large (and perhaps exclusive) preference for Arg over Lys as a gating charge carrier in VSD has not been established. Despite the strong Arg preference, replacing Arg residues with Lys in VSD need not result in attenuation of gating charge (Q) (*e.g.*^9^), indicating that Lys positive charge can be driven across the hydrophobic plug by voltage changes. Likewise, non-titratable positive trimethylammonium adducts on S4 can undergo voltage-driven translocation^10^. In most of these Arg-substituted VSD the properties of the VSD are altered (reduced activation-deactivation kinetics and/or altered Q-V or current (I)-V relationships)^9^,^10^, indicating that the preference for Arg in natural VSD may relate to optimised VSD kinetics associated with a reduced activation barrier for Arg translocation. One suggested possibility is that cation-π interactions between the Arg guanidinium and a highly conserved Phe residue on the S2 helix within the hydrophobic plug of VSD might lower the activation barrier for gating charge transfer^6^. However a role for specific cation-π interactions is contradicted by observations that the conserved Phe can be replaced by planar cyclic non-aromatic side chain analogs in VSD of K^+^ 6 and Na^+^ channels^11^, and that some VS have Leu (rather than Phe) at the equivalent position on S2 (*e.g.* KvAP).

We recently proposed that the large preference for Arg over Lys as a gating charge carrier relates to the particular hydration properties of the two side chain ions, guanidinium for Arg and aliphatic amino for Lys^12^. Mason *et al.*^13^ used neutron diffraction with isotopic substitution (NDIS) to show that the protonated planar guanidinium ion (Gdm^+^) is poorly hydrated (*i.e.* hydrophobic) above and below the molecular plane while retaining in-plane hydration. In molecular dynamics (MD) simulations with forced S4 movement the Arg guanidinium group has been shown to slide/rotate through the voltage sensor plug^14^,^15^,^16^,^17^. The facility of the Arg guanidinium to shed waters above and below the molecular plane may enable a low energy path for the Arg side chain through the hydrophobic plug^12^. In-plane solvation of the guanidinium can be largely maintained by hydrogen bonding to waters and negatively charged side chains sequentially below and above the hydrophobic plug (see Fig. 1). Conversely, the positive charge focused on the terminal amino group of the Lys side chain should be less easily transferred through a hydrophobic environment since the spherical hydration structure of the amino group^18^ cannot easily be maintained during charge transfer^12^.

If the Arg side chain interacts more favourably with hydrophobic amino acids during gating charge transfer, then this should be reflected in significant representation of the Arg guanidinium with hydrophobic amino acids in protein structures. This has been shown to be the case for the well-characterised stacking interaction involving the Arg side chain with aromatic side chains^19^, and early analyses of side chain interactions in proteins are broadly consistent with non-polar side chain interactions with the arginine side chain^20^. Here we describe a systematic analysis of Arg and Lys side chain interactions in the protein data bank. We have examined the distributions of the 19 non-glycine amino acid side chains around the side chain guanidinium of Arg and the aliphatic amino group of Lys. The atom distributions around the Arg side chain are consistent with the hydration structure of Gdm^+^ and support the interpretation that the hydration structure of the Arg side chain underlies its role as a mobile gating charge carrier in membrane voltage sensor domains.

## Results

### Distribution of non-polar side chains around Arg and Lys

Figure 2 illustrates the distribution of carbon atoms of the aliphatic side chain of leucine around the positively charged termini of the side chains of arginine and lysine in the protein data bank. In Fig. 2a,b the carbon atoms within 4.5 Å of the atoms that define the coordinate system of the Arg guanidinium group or the end of the lysine side chain (see Fig. 2d) are represented as small spheres. The atom distributions are quite different for the two positively-charged side chains. For Arg, close Leu side chain carbons partition above and below the plane of the side chain guanidinium, and the Leu atom density extends over and beyond the full guanidinium group. The coordinate space in the plane of the guanidinium group is considerably depleted in Leu side chain density. On the other hand the Leu side chain density around Lys shows little orientational dependence, and the atom density largely surrounds the aliphatic part of the Lys side chain. The marked asymmetry in the non-polar carbon distribution around Arg, with high carbon density lying above and below the guanidinium plane and little in-plane density, is particularly marked if only non-polar carbon atoms within 3.75 Å are shown, as seen for example, in the distribution of Val side chain carbon atoms around Arg (Supplementary Fig. S1).

The results for leucine are illustrated graphically in Fig. 2c in which atom distributions (closest Leu side chain carbon atom within 6 Å of an Arg or Lys side chain) are displayed as polar coordinates according to the coordinate systems defined in Fig. 2d. The θ angle defines the angular deviation from the positive z direction (0, 180°) so that a value of 90° defines Leu carbon atom density in the plane of the guanidinium group (Arg) or the plane defined by the Cδ-Cε-Nz atoms for Lys. The *ψ* angle defines the rotation angle within the xy plane (*i.e.* the plane of the guanidinium group for Arg), with a value of zero when aligned along the x axis (Fig. 2d). The plots reinforce the spatial description of the Leu side chain carbon density of Fig. 2a in showing high aliphatic carbon density above and below the plane of the Arg guanidinium but little in-plane density especially along the direction of the guanidinium NH bonds. For Lys there is little aliphatic carbon density around the side chain amino group, and the atom density is concentrated at angles above ±90° corresponding to Leu side chain atom density that lies around the aliphatic parts (Cβ to Cε) of the Lys side chain.

The distributions of non-polar (aliphatic or aromatic) side chain density around the side chains of Arg and Lys in the protein data bank is broadly similar for the other non-polar side chains (Fig. 3, Supplementary Figs S2–S4). In each case, aliphatic or aromatic non-polar side chain carbon atoms are located largely above and below the plane of the guanidinium group of arginine, the distribution extends over the entire guanidinium group and there is little non-polar carbon density from near neighbor side chains in the plane of the guanidinium. For lysine the near-neighbor non-polar side chain carbon density is spherically-distributed and lies largely around the aliphatic part of the Lys side chain (Fig. 2, Supplementary Figs S3 and S4). In the case of methionine around Arg, sulphur atoms are over-represented in close (<4 Å) in-plane positions compared to the aliphatic carbons of the side chain (Fig. 3b), consistent with hydrogen-bonding with guanidinium NH groups. In addition to the well-characterized stacking of aromatic side chains with the arginine guanidinium group represented by the strong Trp side chain density above and below the guanidinium plane (Fig. 3c and Supplementary Fig. S2)^21^, Trp shows considerable in plane atom density with arginine, especially adjacent to the NH1 guanidinium nitrogen. Inspection of the arrangement of Trp indole side chains in these positions indicates that these represent Arg-Trp cation-π interactions (Supplementary Fig. S5).

### Distribution of polar and charged side chains around Arg and Lys

The non-polar side chain distributions around the Arg guanidinium group are consistent with the interpretation of the hydration structure of the guanidinium cation using neutron diffraction with isotopic substitution (NDIS)^13^, which indicates that guanidinium is poorly-solvated (hydrophobic) above and below the molecular plane despite carrying a delocalized positive charge. The distribution of *polar* groups around the Arg side chain in the protein data bank is similarly polarised with hydrogen-bonding polar atoms lying largely in the plane of the side chain guanidinium group as illustrated for the carboxyl O atoms of Asp around Arg in Fig. 4. The Asp carboxylate oxygens in the vicinity of the guanidinium group largely occupy the space around the guanidinium that lacks hydrophobic atom density (compare Fig. 4a,c with the Arg-Leu density in Fig. 2). The Asp carboxylate oxygens are spherically distributed around the lysine amino terminus, although fixing the lysine side chain in the coordinate system of Fig. 2d generates a “propeller-like” distribution with the oxygens clustered with respect to the three directly-bonded amino nitrogen hydrogen atoms (Fig. 4c). As in the case of the Asp carboxylate distribution around the Arg guanidinium group, the polar oxygens occupy the space around the end of the lysine side chain that lacks hydrophobic carbon atom density (compare Fig. 4b,c with the Lys-Leu density in Fig. 2). These observations are in accordance with a previous analysis of carboxylate side chain distributions around Arg and Lys in the context of salt bridge geometries in proteins^22^.

Segregated atom distributions around the guanidinium group of Arg apply for the other polar amino acid side chains. As for aspartic acid, the side chain amide oxygens of asparagine occupy axial hydrogen bonding positions in the plane of the guanidinium group (Fig. 5a). There is somewhat more Asn amide O atom density above and below the guanidinium plane than observed for Asp (compare with Fig. 4a) as well as significant amide nitrogen density above and below the guanidinium. This arises from “stacking” of planar Asn side chain amide groups with the planar Arg guanidinium as illustrated in Supplementary Fig. S6. Amino acid side chains having both polar and non-polar side chain groups also show strong atom segregation, as illustrated for the threonine side chain distribution in Fig. 5b. The Thr side chain Cβ and Cγ largely occupy space above and below the Arg guanidinium plane whereas the side chain hydroxyl oxygen atoms are clustered in or near the plane of the guanidinium group. The carbon atom density that lies in or near the plane of the Arg guanidinium lies *outside* the Thr side chain oxygen density and corresponds largely to carbon atoms of Thr side chains whose Oγ atoms occupy in-plane hydrogen-bonding positions. The full sets of distributions of side chain heavy atoms of each of the 19 non-glycine amino acids around the side chains of arginine and lysine in the protein data bank are shown in Supplementary Figs S2 and S3.

### Arginine side-chain environments in proteins

The distributions of non-polar and polar atoms around the side chains of Arg and Lys in Figs 2, 3, 4, 5 and Supplementary Figs S1–S6 correspond to *populations* of interactions represented in the protein data bank, rather than individual arginine side-chain environments. However arginine side chains can be found in the protein data bank in environments in which non-polar side chains are the only closely positioned residues above *and* below the molecular plane of a side chain guanidinium; a number of examples are illustrated in Supplementary Fig S7. In these examples the hydrogen bonding potential of the Arg side chain is satisfied by hydrogen bonding with oxygen atoms (of water molecules, side chains carboxylates and backbone carbonyls) that lie in or near the plane of the guanidinium group.

Observation of Arg side chains in similar environments within crystal structures of VSD are not necessarily expected since translocation of guanidinium side chains through the hydrophobic plug should correspond to transition states between conformations in which the gating charge lies sequentially below or above the plug. These states are less likely to be captured in crystal structures and indeed VSD Arg guanidinium groups are generally located below or above the hydrophobic plug charge-paired with Asp or Glu side chain carboxylate groups. However, one example that does conform to this motif is R229 in the activated-state structure of the *Ciona intestinalis* voltage sensitive phosphatase Ci-VSP R217E mutant^8^ (Fig. 6a). The R229 side chain guanidinium in this structure is sandwiched between Ile residues (I126 and I190) while making in-plane electrostatic/hydrogen bond interactions with D186 carboxylate and the F161 aromatic (cation-π interaction similar to those illustrated for in-plane Arg guanidinium Trp aromatic cation-π interactions illustrated in Supplementary Fig. S5). More generally, Arg environments in VSD correspond to the theme of in-plane hydrogen bond/solvation of the side chain guanidine with, in some cases, a bulky non-polar side chain lying above or below the guanidinium plane (*e.g.* R201 in the mouse proton channel chimera structure PDB: 3WKV^23^; Fig. 6b).

## Discussion

The interaction of Arg side chains with aromatic groups in protein structures is well established^19^,^21^ with a preference for stacking between the aromatic ring and the Arg side-chain guanidinium. Likewise, Harms *et al.* have shown that the arginine guanidinium retains its charge even within apparently hydrophobic environments in proteins^3^, and some interactions of Arg with non-polar side chains^20^ and ligands in drug-protein complexes^24^, have been described. Our results demonstrate that the distribution of polar and non-polar side chains around the Arg guanidinium in proteins strongly reflects the hydration structure of the guanidinium ion (Gdm^+^) determined using NDIS^13^; the ion readily sheds hydration waters above and below the molecular plane but retains in-plane hydrogen bonding. The surfaces of the guanidinium ion are therefore hydrophobic and the ion interacts favourably with all non-polar (especially planar^25^,^26^) surface in proteins that lies above and below the plane of the guanidinium (Figs 2, 3, 5b, 6 and Supplementary Figs S1, S2, S5–S7). However Gdm^+^ forms hydrogen bonds with water molecules in the molecular plane^13^,^18^. In proteins the in-plane hydrogen bonding potential of the Arg guanidinium is satisfied largely by oxygen hydrogen bond acceptors of the carboxylate and hydroxyl containing amino acids (Figs 4, 5, 6 and ref. 22), water molecules (*e.g.* Supplementary Fig. S7 and ref. 18) and main chain amide carbonyl groups^21^, although the latter were not examined here. The strong segregation of in-plane polar and out-of-plane non-polar surface is likely to underlie the properties of Gdm^+^ as a protein denaturant^13^,^27^,^28^, and its role (as the guanidinium group of Arg) as a mobile charge carrier in VSD of ion channel proteins and other voltage-responsive membrane proteins^8^,^23^ as described below.

The appearance from the spatial distribution figures is for the arginine guanidinium to be sandwiched within a non-polar “stack”, with polar groups “solvating” the hydrogen bonding NH groups in the molecular plane. Although these figures represent the superposition of large numbers of examples from the protein data bank, inspection of individual examples demonstrates that the Arg side chain can be found in such configurations (Supplementary Fig. S7). The implication is that these and similar environments^3^ constitute low energy or energetically neutral configurations for arginine side chains in proteins. The distributions of amino acid side chains around the Lys side chain are quite different, with non-polar carbon density localized largely over the non-polar part of the side chain (Cβ-Cε carbons) (Fig. 2), and a radial distribution of hydrogen bonding groups around the terminal amino group (*e.g.* Fig. 4b), as previously described for the distributions of crystal structure waters around the lysine side chain amine^18^.

**Figure 1:**
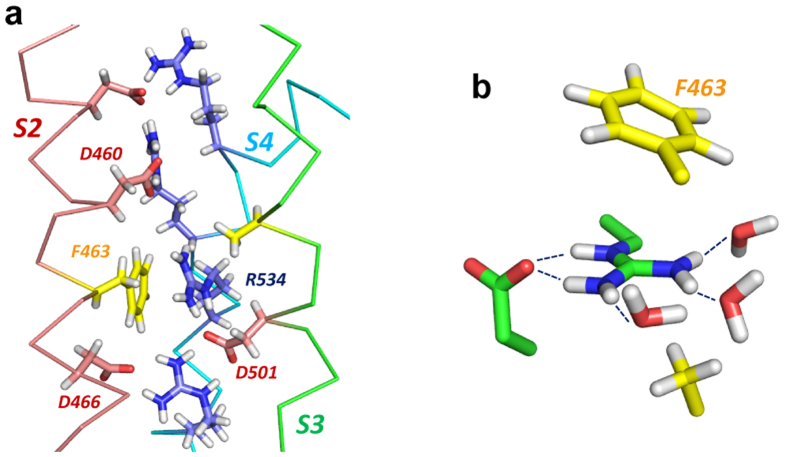
S4 arginine side chains slide through a hydrophobic plug to transfer gating charge in voltage sensing domains. (**a**) Snapshot (80 ns) of the central region of a voltage sensor domain of a hERG K^+^ ion channel model during molecular dynamics simulation in a hydrated POPC bilayer^43^. The S2, S3 and S4 helices of the voltage sensor are annotated; the S1 helix has been removed for clarity. In this simulation the R534 side chain initially interacts with D466, but moves across the hydrophobic plug (indicated by yellow non-polar side chains) to interact with the D460 side chain^12^. (**b**) During this transition the guanidinium group maintains in-plane solvation with water and Asp carboxylate oxygen atoms while moving between non-polar side chains. Dotted lines indicate hydrogen bond distances ≤2.0 Å. See ref. 12 for details.

These observation help to explain the strong preference of Arg as a carrier of voltage-responsive gating charge in membrane voltage sensor domains. In response to voltage changes the gating charge carriers of voltage sensors must move through a non-polar “plug” while maintaining charge solvation. Molecular dynamics simulations suggest that this can be achieved with the guanidinium group of Arg, but not easily with Lys^12^. The similarities in the environment of the Arg side chain in protein crystal structures in Supplementary Fig. S7, and the environment of an arginine side chain as it moves through the VS hydrophobic plug in a hERG potassium channel model simulation (Fig. 1^ ^12), are marked. Likewise, the atom distributions around Arg and Lys side chains from molecular dynamics simulations of VSDs are similar to those found from inspection of crystal structures (see Supplementary Fig. S8). Despite some uncertainty about the extent to which protein side chain charges should be re-scaled in MD simulations to match experimental measures of charge pairing interactions (*e.g*.^15^,^29^,^30^), these similarities in atom distributions indicate that the general hydration structures and charge-pairing geometries involving Arg and Lys side chains can be well-represented in simulations.

The interpretation that it is the hydration structure of guanidinium that underlies the function of arginine as a mobile charge carrier in VSD, helps to resolve some uncertainty about the nature of the VS hydrophobic plug across which the membrane electric field is focused, and in particular the role of the Phe residue that lies within the plug on the S2 helix of most VSD (*e.g.* F463 of hERG in Fig. 1). Specific interactions (like cation-π interactions) involving Arg gating charges and conserved aromatic residues within the plug are not important for efficient gating charge transfer since gating pore aromatics can be replaced by non-aromatic planar side chains and/or bulky aliphatic side chains^6^,^11^,^31^,^32^. Non-polar side chains in the hydrophobic plug are absolutely required for functional VSD, but it is the overall hydrophobicity or side chain volume that seems to be important for these residues^33^. These observations can be accommodated in a model in which it is the poorly-solvated surfaces of the arginine guanidinium that allows it to interact with generalized non-polar surface within the VS hydrophobic plug, together with its ability to retain charge solvation as it traverses the electric field, that underlie the preference for arginine as a gating charge carrier (see Fig. 7). A dominant role for Arg might also relate to a requirement for the side chain to occupy the hydrophobic plug at resting (activated or non-activated) states to maintain a seal within the VS (see *e.g*.^34^).

In addition to disfavoring Lys as a gating charge carrier, the preferred atom distributions around Lys may positively favor the occupation by Lys of specific “docking” sites in voltage sensor domains. For example, a charge transfer centre^6^ occupied by Lys-302 below the hydrophobic plug of the Kv1.2/2.1 chimera voltage sensor in the depolarized (VS “up”) state (Fig. 7d)^35^, is well matched to the spherical solvation/charge-pairing and non-polar interaction requirements of the Lys side chain^12^. As the S4 helix of voltage sensors responds to changes in membrane voltage, a discrete set of gating charges moves across the hydrophobic plug; however additional non-transferred charges seem to be required at positions N- and C-terminal to the transferred arginines to satisfy charge-pairing requirements within the voltage sensor. In circumstances where Lys plays this role, the trapping of the Lys side chain in the charge transfer center site^6^ may allow the VS to “click” into a conformationally-discrete S4-up state under depolarizing potential^12^.

Finally, the hydration properties of the guanidinium group likely underlies the high guanidinium omega current conductance in ion channel mutants carrying truncated S4 side chains at wild-type Arg positions^36^. Truncation of a VS Arg side chain leaves a voltage-dependent “guanidinium-sized” hole through which a guanidinium ion can move like a coin through a slot. Notably, the other side chain likely to be carried through the “guanidinium slot” in the hydrophobic plug of native VSDs is glutamine, which is found, for example, within the expected translocated region of the S4 helix of KCNQ channels (Kv7) (*e.g.* ref. 37). The side chain of glutamine is also a planar π-bonded group that has non-polar character above and below the amide plane^38^. It thus shares some of the properties of the Arg guanidinium group that affords a low energy path through the VSD hydrophobic plug, allowing, in the case of KCNQ channels, a voltage sensor with modulated response to voltage arising from translocation of an uncharged side chain across the membrane electric field.

## Methods

### Analysis of PDB files; Parsing

A list of PDB chains culled for 30 percent sequence identity, a minimum resolution of 1.6 Å and R factor of 0.25 generated on the 12th of December 2013 was obtained from the Pisces web server^39^. The full set of PDB files from the same date was downloaded from the RCSB protein data bank^40^, resulting in 3,450 individual protein chains. A python script was written to parse the PDB chains corresponding to the Pisces output so that each arginine and lysine residue and any amino acid within 5 Å was exported to a separate PDB-like file (one for each interacting pair; a single arginine might have multiple associated PDB-like files, for example). The coordinate system for each file was transformed so that the functional groups of either the arginine or lysine were overlaid. This was achieved by translating the CZ carbon of arginine or the NZ nitrogen of lysine back to the origin, and then using the Kabsch algorithm to align the (CZ, NH1, NH2) coordinates of arginine with ([0.0, 0.0, 0.0], [0.5, 0.87, 0.0], [0.5, −0.87, 0.0]), and the (NZ, CE, CD) coordinates of lysine with ([0.0, 0.0, 0.0], [−1.0, 0, 0.0], [−1.34, 0.94, 0.0]), using the linear algebra and dot product methods found in the NumPy package^41^. Only side chains were saved in the resulting file, and hydrogen atoms were not outputted. The numbers of “interactions” between Arg or Lys and the 19 non-glycine amino acid side chains are collated in Supplementary Table S1 and Supplementary Fig. S9.

### Visualisation

**Figure 2:**
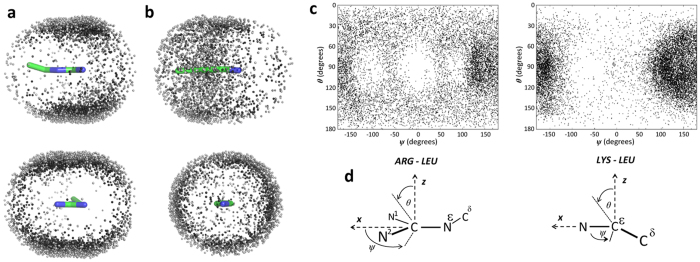
Distributions of leucine side chain carbon atoms around the side chains of arginine and lysine in the protein data bank. (**a,b**) Position of Leu side chain carbon atoms within 4.5 Å of (**a**) the NE, CZ, NH1 and NH2 atoms of Arg, or (**b**) the CD, CE or NZ atoms of Lys in the protein data base shown from the side (top) or front (bottom). In each case the figures show a selection of 4000 atom pairs from the total set of atom pairs identified. Leu carbon atom densities vary from light to dark grey for atoms within 4.5 Å to 3.75 Å, respectively, of the specified side chain atoms. (**c**) Leu side chain carbon distributions represented according to the coordinate systems (**d**) below the plots. The y axis is the angle of rotation from the z direction into the x – y plane (*i.e.* 90° corresponds to Leu side chain carbon atoms in the x – y plane and 180° corresponds to Leu carbon atoms in the -z direction). The x axis corresponds to the rotation around z where 0° defines Leu carbon atoms oriented along x in each coordinate system. Each point corresponds to a Leu side chain carbon atom within 6 Å of the NE, CZ, NH1 or NH2 of arginine (left) or the CD, CE or NZ of Lys (right).

**Figure 3:**
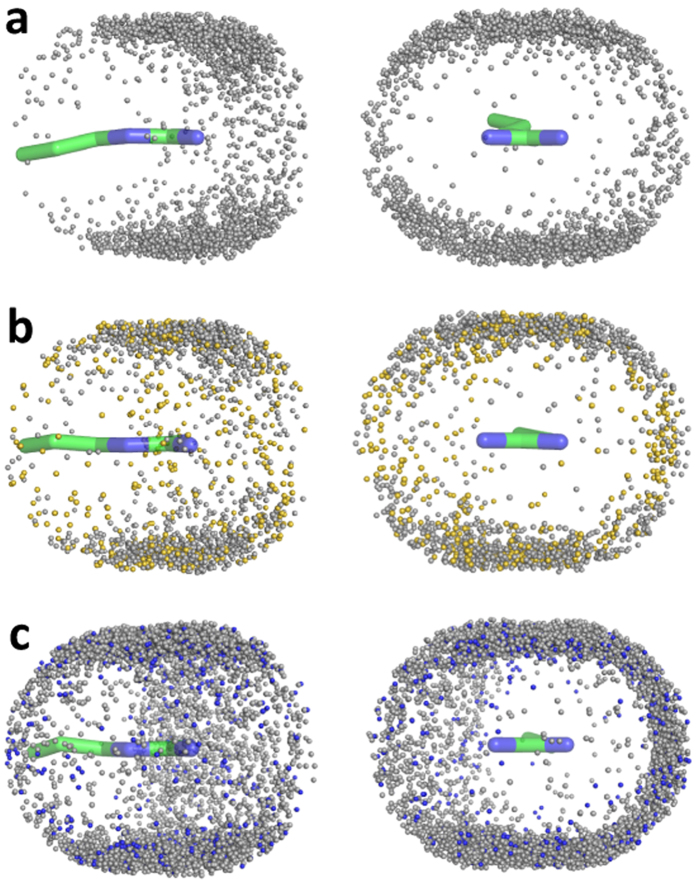
Distributions of non-polar side chain atoms around arginine side chains. Side chain carbons of (**a**) alanine, (**b**) methionine and (**c**) tryptophan, are grey spheres. Met side chain sulphur (SD) atoms are yellow spheres; Trp side chain NE1 nitrogen atoms are blue spheres.

**Figure 4:**
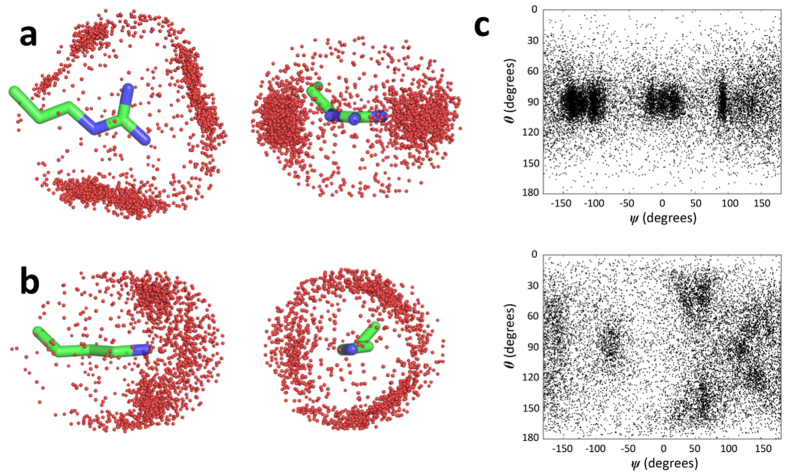
Distributions of aspartic acid side chain carboxylate oxygen atoms around the side chains of arginine and lysine in the protein data bank. (**a**) Side chain carboxylate oxygens within 3.5 Å of the side chain NE, CZ, NH1 or NH2 atoms of the arginine side chain. (**b**) Equivalent aspartic acid carboxylate oxygens within 3.5 Å of the CD, CE or NZ atoms of the lysine side chain. (**c**) Distribution of side chain carboxylate oxygen atoms of aspartic acid around the side chains of arginine and lysine according to the coordinate systems shown in Fig. 2d. Each point corresponds to an Asp side chain oxygen atom within 6 Å of the NE, CZ, NH1 or NH2 of arginine (top) or the CD, CE or NZ of Lys (bottom).

**Figure 5:**
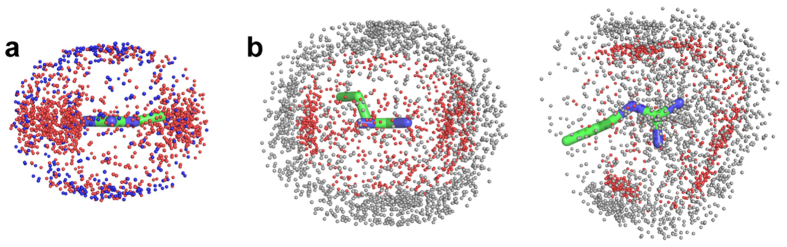
Distributions of asparagine and threonine side chain heavy atoms around the arginine side chain. (**a**) Side chain amide oxygen (red) and nitrogen (blue) atoms of asparagine within 3.5 Å of the side chain NE, CZ, NH1 or NH2 atoms of arginine. (**b**) Distributions of side chain hydroxyl oxygen (red) and carbon atoms (grey) of threonine within 3.5 Å (oxygen) or 4 Å (carbon) of the NE, CZ, NH1 or NH2 atoms of arginine.

**Figure 6:**
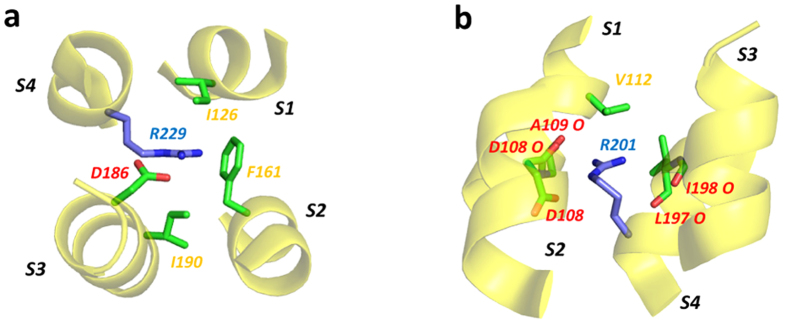
Selected environments of S4 arginine side chains in voltage sensor domains. (**a**) R229 in the VSD of the activated-state mutant (R217E) of the voltage-sensitive phosphatase Ci-VSP (PDB: 4G7V)^8^. The R229 side chain guanidinium is “sandwiched” between the aliphatic side chains of I126 and I190, and makes in-plane polar interactions with the D186 carboxylate and the F161 aromatic ring. (**b**) R201 in the proposed resting state mouse Hv1 chimera proton channel/VSD structure (PDB:3WKV)^23^. The side chain guanidinium group lies within a cluster of in-plane carboxylate and backbone carbonyl oxygens with an aliphatic side chain (V112) lying above the plane of the guanidinium.

**Figure 7:**
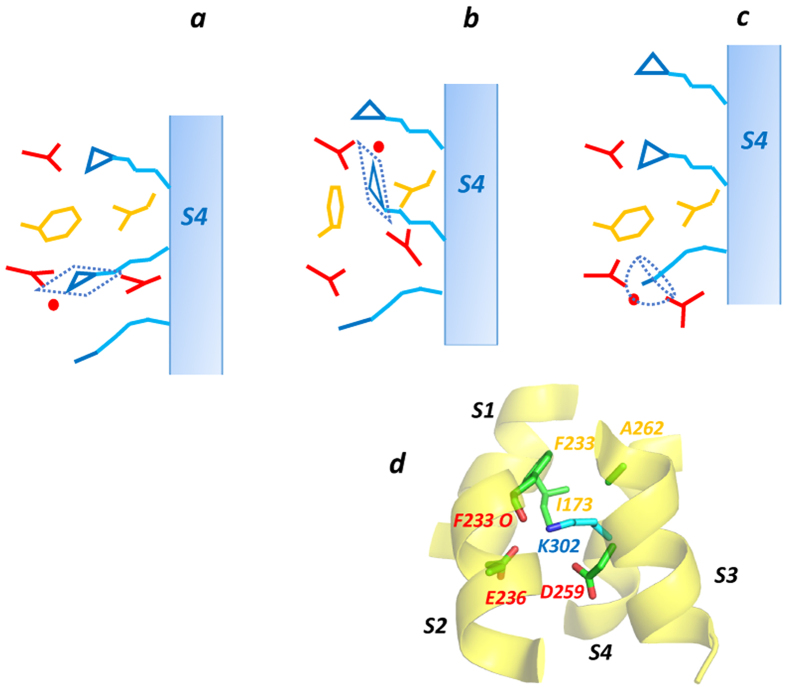
Schematic of movement of an S4 arginine side chain through the hydrophobic plug of a voltage sensor domain. (**a**) An Arg side chain guanidinium (blue triangles) lies in the “charge transfer center” below the hydrophobic plug (yellow side chains) solvated by carboxylate and water oxygens (red) that occupy in-plane positions around the guanidinium as found in protein structures (Figs 4 and 6). In a voltage-driven transition through the plug (**b,c**) the guanidinium finds a low energy path between non-polar side chains while retaining in-plane interactions with carboxylate and water oxygens as observed in molecular dynamics simulations (see Fig. 1). The spherical distribution of carboxylate and water oxygens around the Lys amino group (Fig. 4) and non-preferred interaction of the amino group with aliphatic side chains (Fig. 2) limits the ability of the Lys amino group to carry charge through the hydrophobic plug. (**d**) A Lys side chain occupies the “charge transfer center” that lies below the hydrophobic plug of the Kv1.2/2.1 chimera channel VSD (PDB:2R9R)^35^ with the amino group solvated by carboxylate and backbone carbonyl oxygens in a “propeller” arrangement (see Fig. 4c), and aliphatic side chains interacting with the aliphatic part (Cβ-Cε) of the side chain. Molecular dynamics simulations indicates that is a stable environment for a Lys side chain^12^.

The distribution of atoms of neighbouring residues around the central residue (Arg or Lys) was visualised in two ways. First, the coordinates of the closest non-hydrogen atom of the non-central residue were expressed as spherical polar coordinates, θ (angle made with the positive z axis), and *ψ* (angle around the z-axis from the positive x-axis), with the distance cut-off omitted. The x and y axes lie in the plane of the guanidinium group of Arg or the plane defined by the CD, CE, NZ atoms the Lys side chain, respectively (see Fig. 2d). This allowed for the distributions to be expressed in two dimensional density plots. Note that the choice of atom placed at the origin does affect the distribution of spherical polar coordinates, and that the areas in the extremes of the plots (very high and very low values of θ) represent smaller volumes in Cartesian space than the values closer to 90 degrees. Second, a selection of the PDB-like files (either all of the files, or the first 4000 in cases where the number of files was greater than 4000) containing individual amino acid pairs was loaded into Pymol^42^, and the distributions of selected atoms was displayed by making Pymol selections with chosen distance separation cutoffs. In cases where the number of files for a particular interaction pair was much greater than 4000, a comparison of the first 4000 files and the last 4000 files showed that file selection did not affect the general distribution of amino acid side chains around the target (Arg or Lys) side chain (see, for example, Supplementary Fig. S1).

## Additional Information

**How to cite this article**: Armstrong, C. T. *et al.* Arginine side chain interactions and the role of arginine as a gating charge carrier in voltage sensitive ion channels. *Sci. Rep.* **6**, 21759; doi: 10.1038/srep21759 (2016).

## Supplementary Material

Supplementary Information
