# Supplementary material for: Arginine side chain interactions and the role of arginine as a gating charge carrier in voltage sensitive ion channels
Source: Sci Rep. 2016 Feb 22;6:21759. doi: 10.1038/srep21759 (PMC4761985; doi:10.1038/srep21759)
Supplement: Supplementary Information [file srep21759-s1.pdf]

Arginine side chain interactions and the role of arginine as a mobile charge carrier in voltage sensitive ion channels

*Craig T. Armstrong†, Philip E. Mason‡, J. L. Ross Anderson† and Christopher E. Dempsey†**

† School of Biochemistry, Bristol University, Bristol BS8 1TD, U.K.

‡ Institute of Organic Chemistry and Biochemistry, Academy of Sciences of the Czech Republic and Center for Biomolecules and Complex Molecular Systems, 16610 Prague 6, Czech Republic

Supplementary Information

Figure S1: Distribution of Val side chain carbon atoms within 3.75 Å of Arg guanidine group in the protein data bank.

Figure S2: Scatter plots of specified amino acid side chains around the Arg guanidine group in the protein data bank.

Figure S3: Scatter plots of specified amino acid side chains around the Lys aliphatic amino group in the protein data bank.

Figure S4: Example of distribution of hydrophobic side chain atoms around the Lys aliphatic amino side chain.

Figure S5: Identification of in-plane cation-π interactions between Arg guanidine group and Trp indole group.

Figure S6: Identification of stacking interactions between the Arg guanidine group and the side chain amide group of Asn.

Figure S7: Selected examples of partially buried arginine side chains in protein structures in which an Arg side chain is "sandwiched" between non-polar side chains with hydrogen bonding donor oxygen atoms near in-plane positions.

Figure S8: Comparison of side chain and water oxygen distribution around Lys amine and Arg guanidine groups from MD simulations of voltage sensitive ion channel models and from inspection of the protein data bank.

Figure S9: Interaction frequencies for Arg and Lys side chain interactions in the protein data bank, and frequencies scaled for relative amino acid composition in the PDB.

Table S1: Numbers of "interacting" pairs for each of the 19 non-glycine amino acid side chains with the side chains of Arg and Lys.

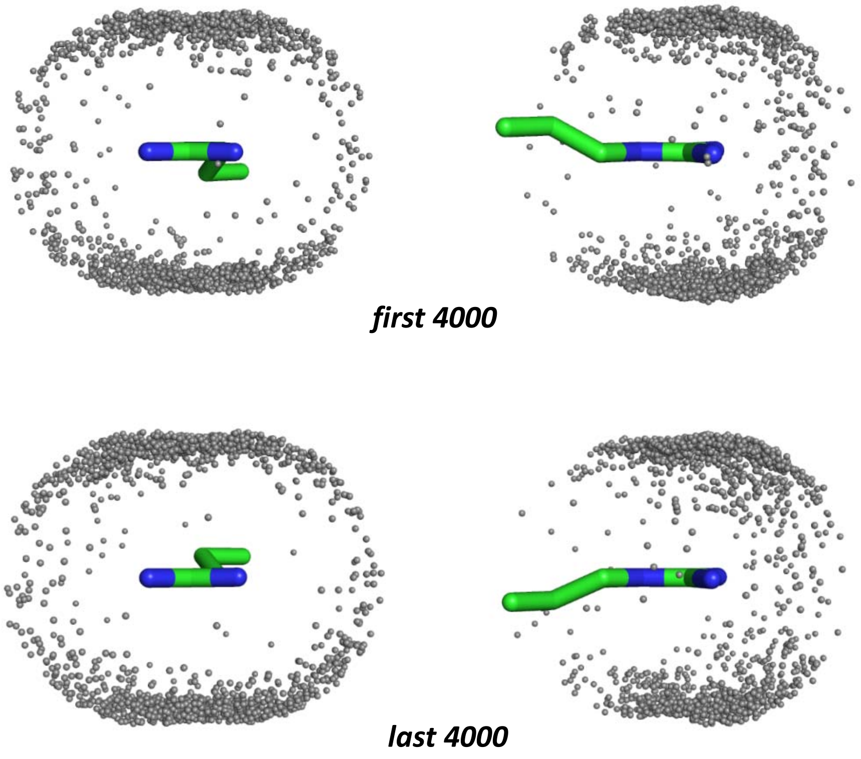

Figure S1: Distribution of Val side chain C atoms within 3.75 Å of any of the Nε, CZ, NH1 and NH2 guanidine side chain atoms of Arg in the protein data bank. A total of 7537 Arg-Val interactions were extracted from the culled PDB data set. The top and bottom pairs show the distributions within the first and last 4000 of the entries, respectively.

Figure S2: Scatter plots of amino acid side chain atoms within 6 Å of the Arg guanidine group in the protein data bank. The coordinate system defining distributions around the Nε, CZ, NH1, NH2 atoms is illustrated in Figure 2 of the main paper.

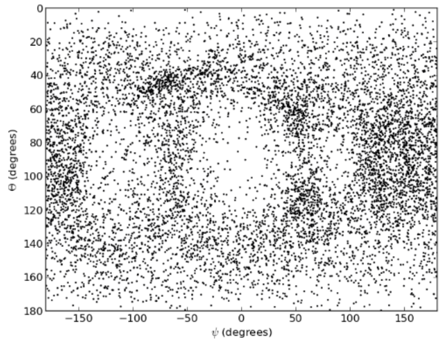

Arg-Ala

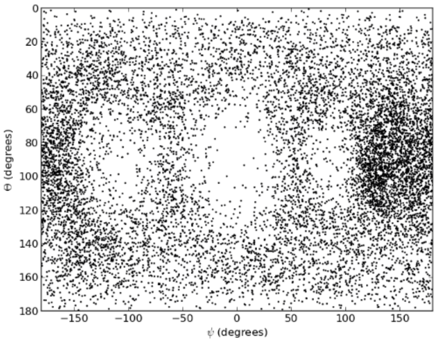

Arg-Val

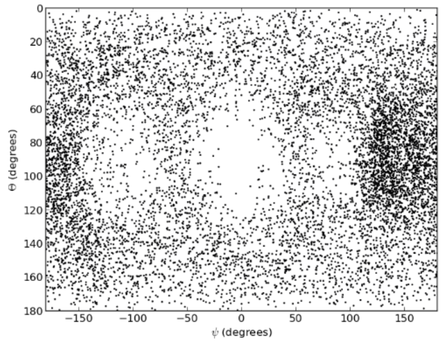

Arg-Leu

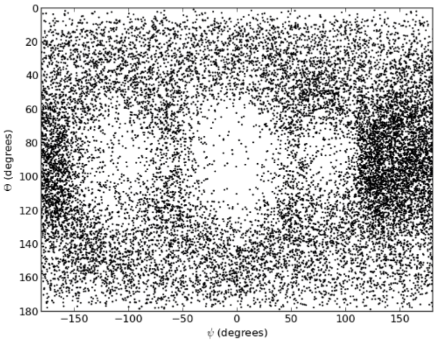

Arg-Ile

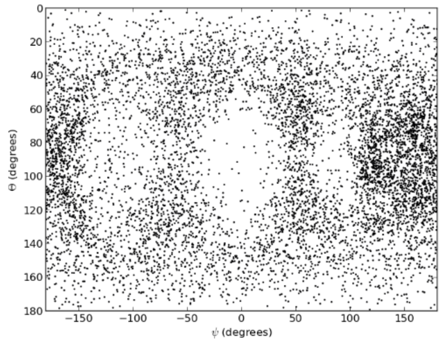

Arg-Pro

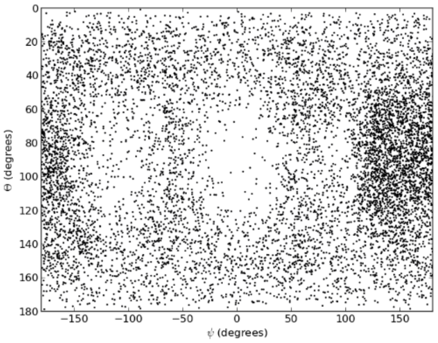

Arg-Phe

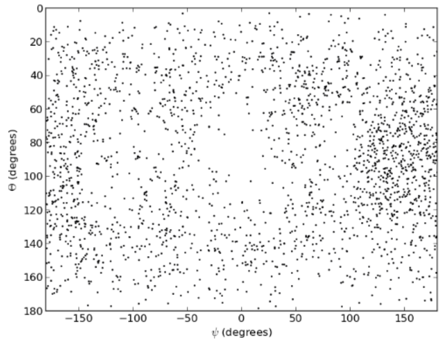

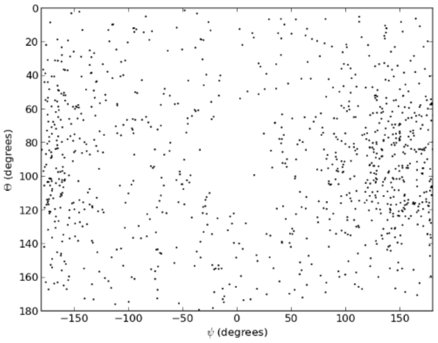

Arg-Met-C

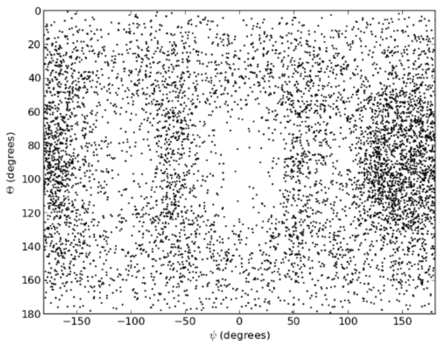

Arg-Met-S

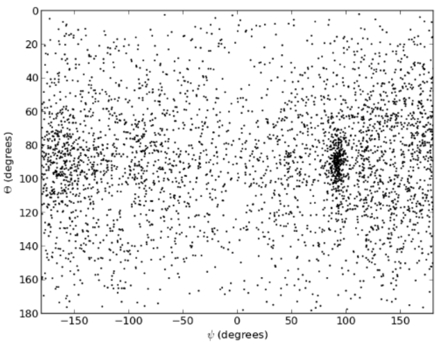

Arg-Tyr-C

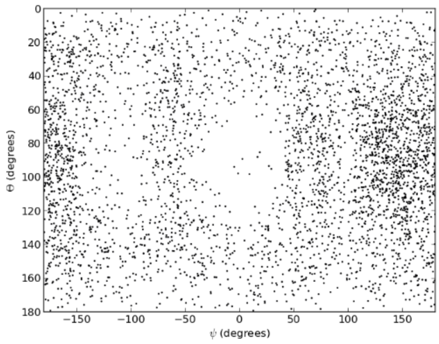

Arg-Tyr-O

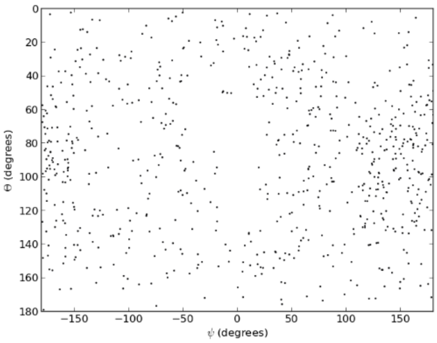

Arg-Trp-C

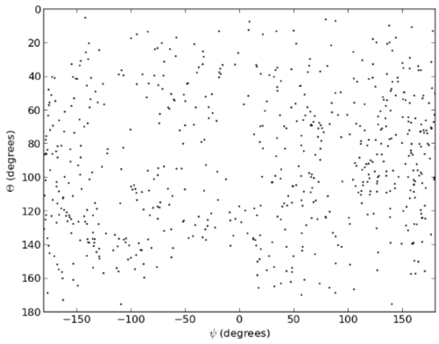

Arg-Trp-N

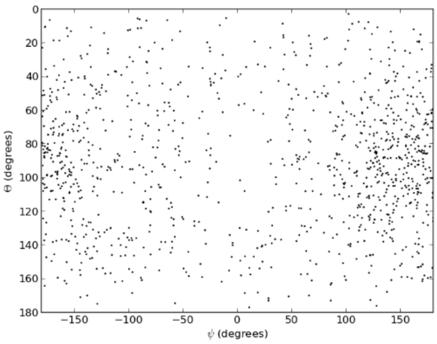

Arg-Cys-C

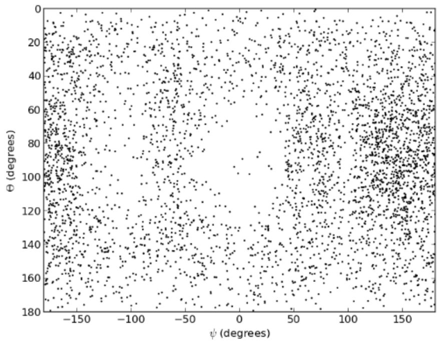

Arg-Cys-S

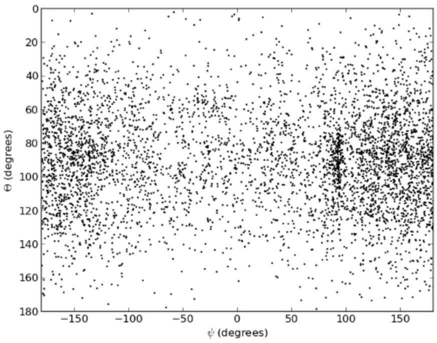

Arg-Thr-C

Arg-Thr-O

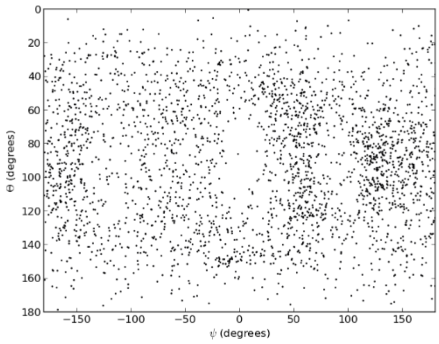

Arg-Asp-C

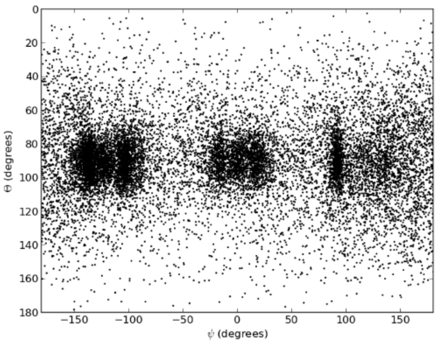

Arg-Asp-O

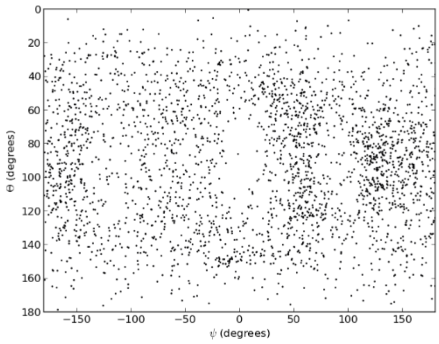

Arg-Glu-C

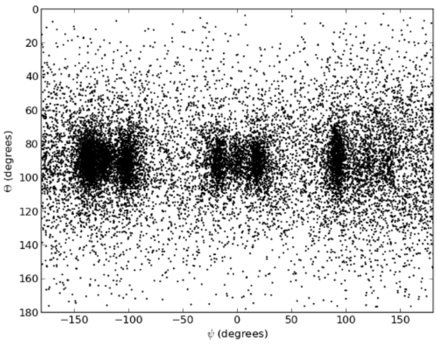

Arg-Glu-O

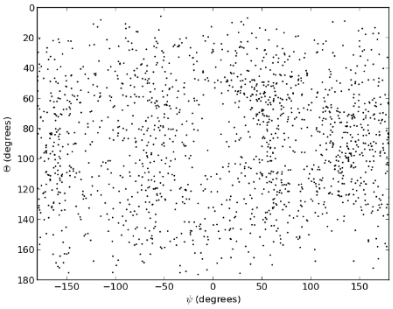

Arg-Asn-C

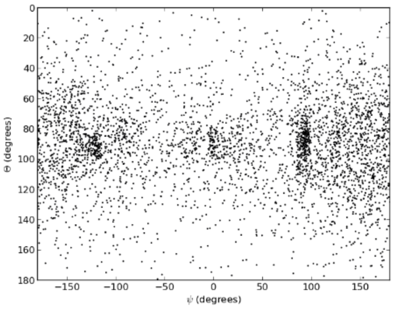

Arg-Asn-O

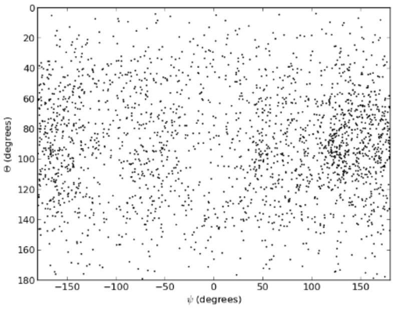

Arg-Asn-N

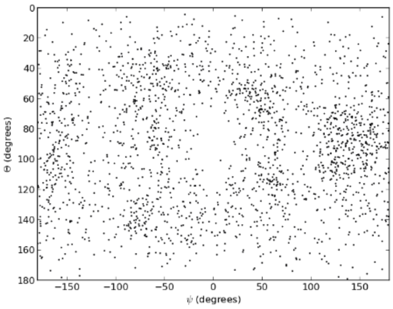

Arg-Gln-C

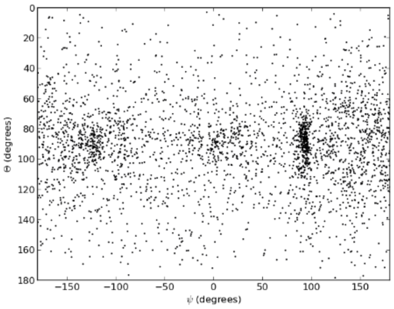

Arg-Gln-O

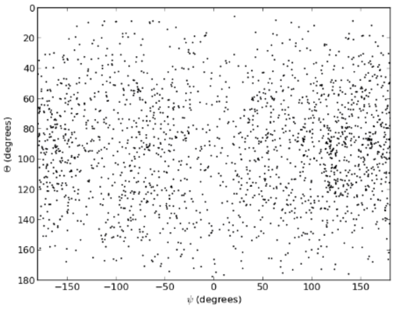

Arg-Gln-N

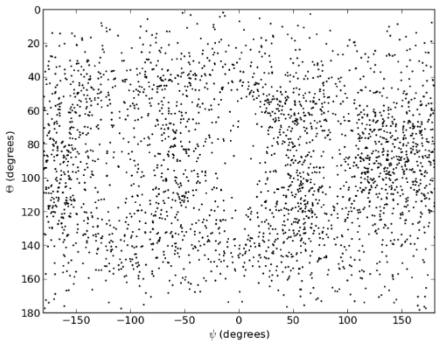

Arg-Ser-C

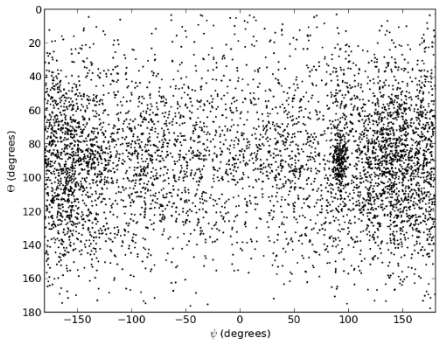

Arg-Ser-O

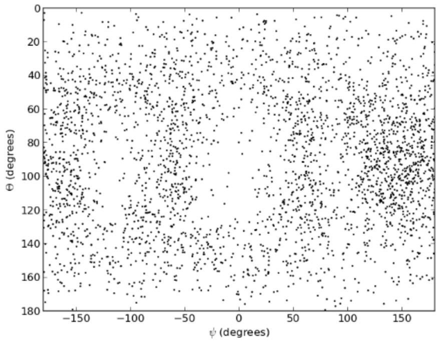

Arg-His-C

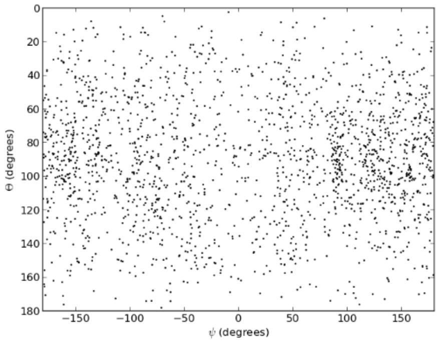

Arg-His-N

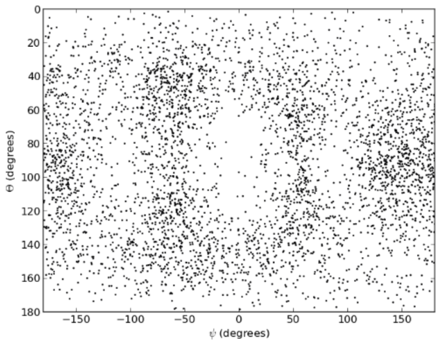

Arg-Lys-C

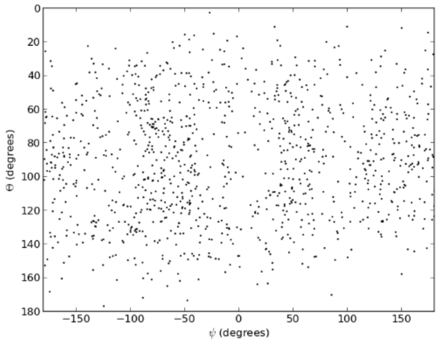

Arg-Lys-N

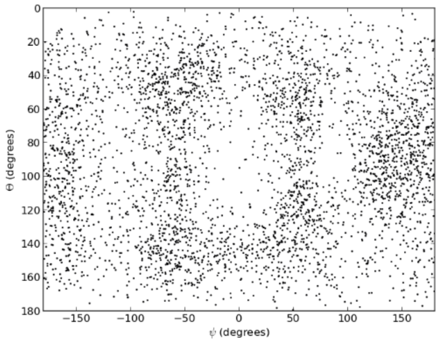

Arg-Arg-C

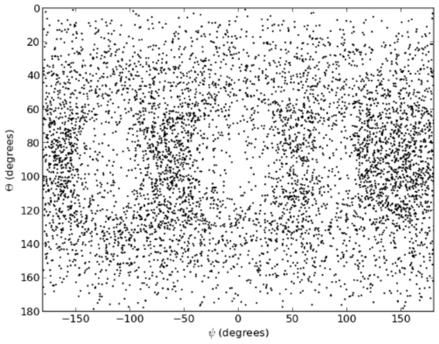

Arg-Arg-N

Figure S3: Scatter plots of amino acid side chain atoms within 6 Å of the Lys aliphatic amino group in the protein data bank. The coordinate system defining distributions around the $C\gamma$, $C\delta$, $N\varepsilon$ atoms is illustrated in Figure 2 of the main paper.

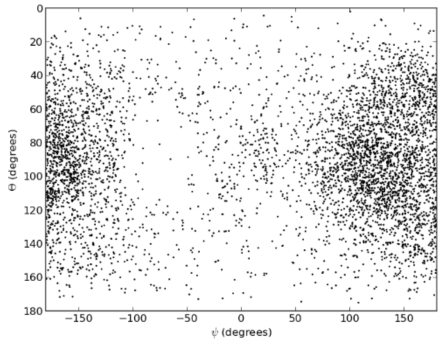

Lys-Ala

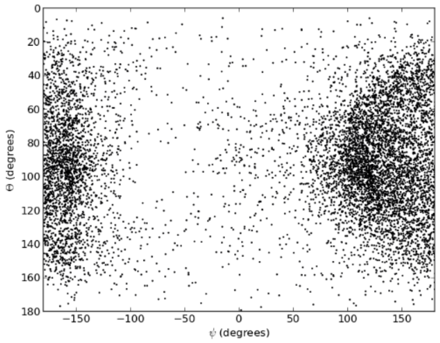

Lys-Val

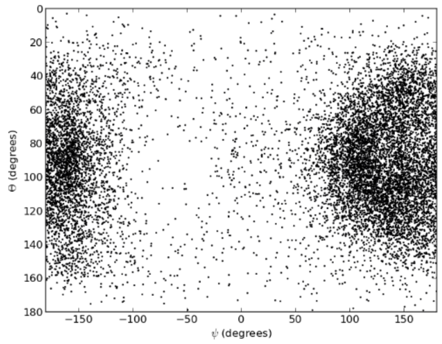

Lys-Leu

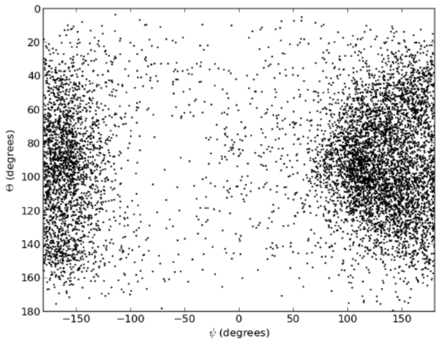

Lys-Ile

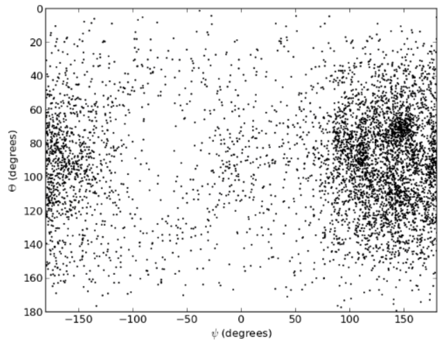

Lys-Pro

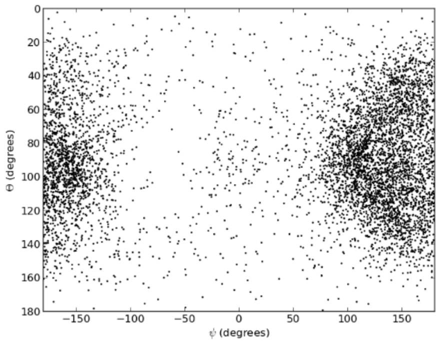

Lys-Phe

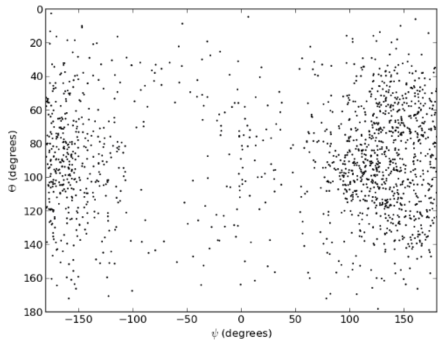

Lys-Met-C

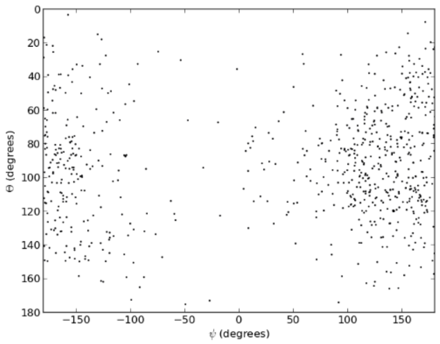

Lys-Met-S

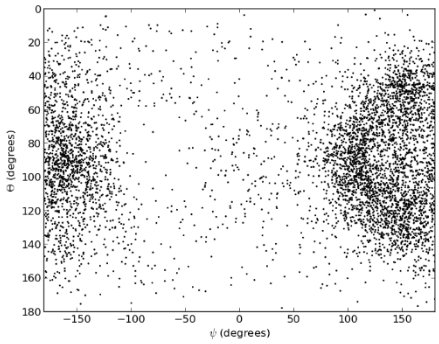

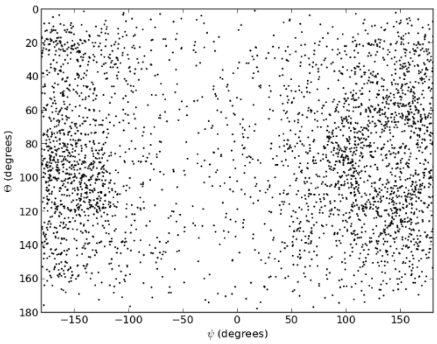

Lys-Tyr-C

Lys-Tyr-O

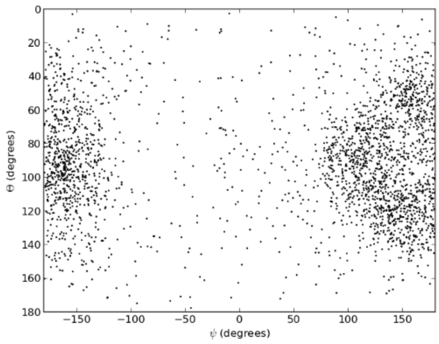

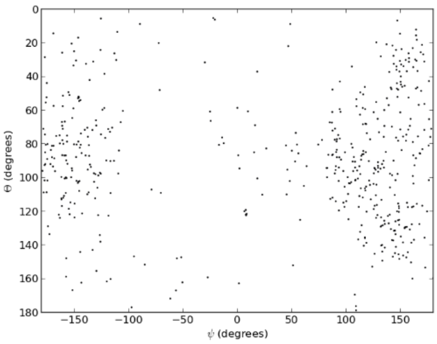

Lys-Trp-C

Lys-Trp-N

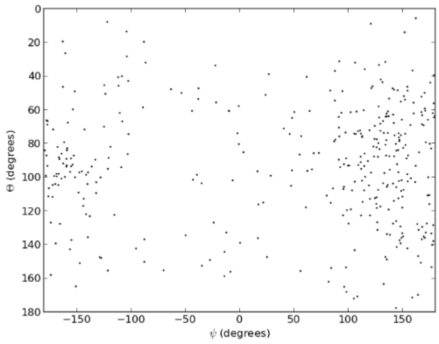

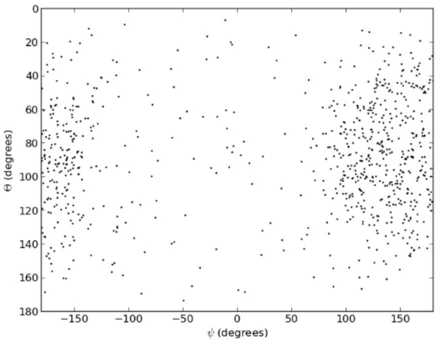

Lys-Cys-C

Lys-Cys-S

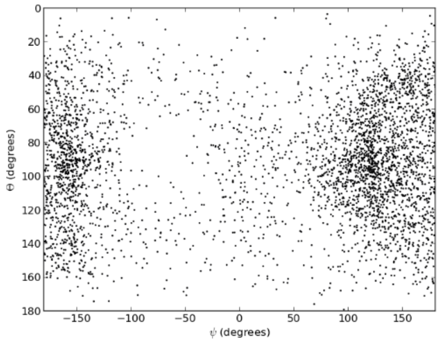

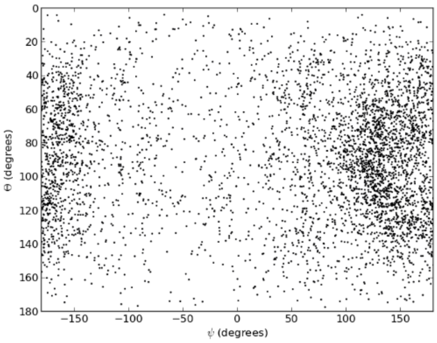

Lys-Thr-C

Lys-Thr-O

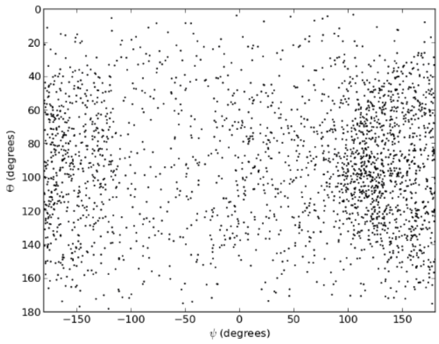

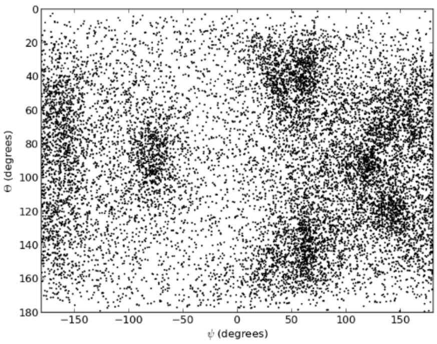

Lys-Asp-C

Lys-Asp-O

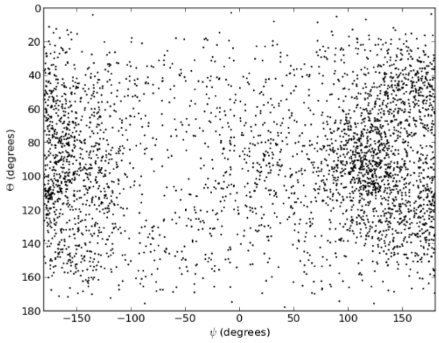

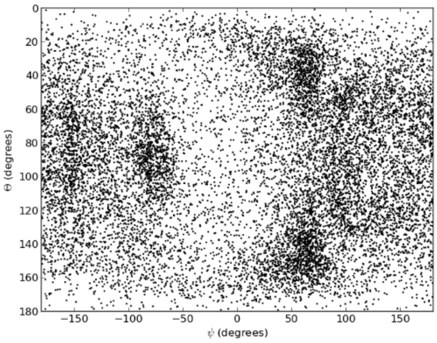

Lys-Glu-C

Lys-Glu-O

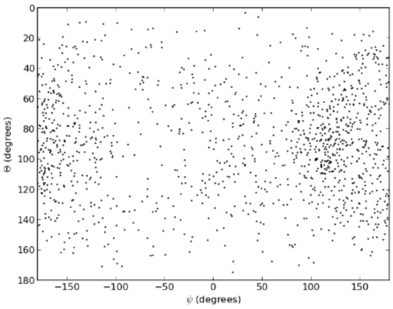

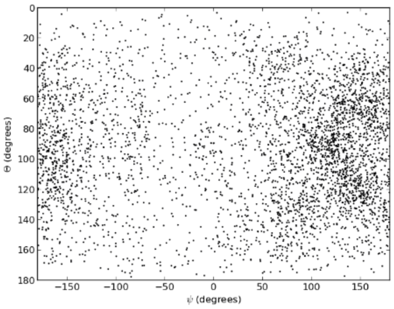

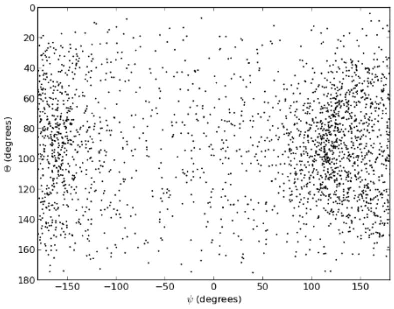

Lys-Asn-C

Lys-Asn-O

Lys-Asn-N

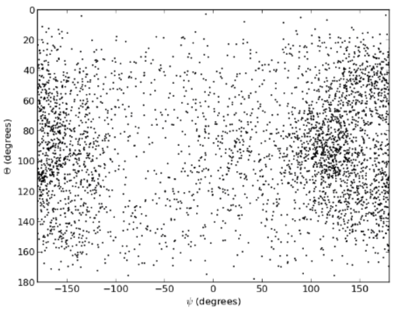

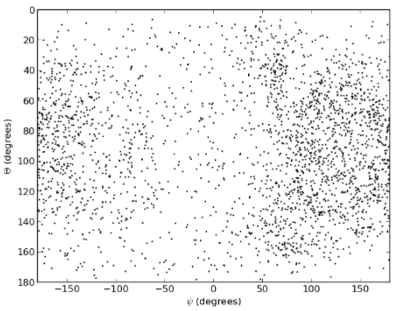

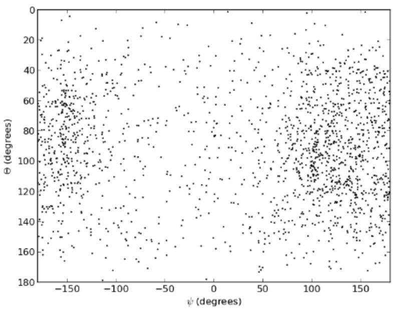

Lys-Gln-C

Lys-Gln-O

Lys-Gln-N

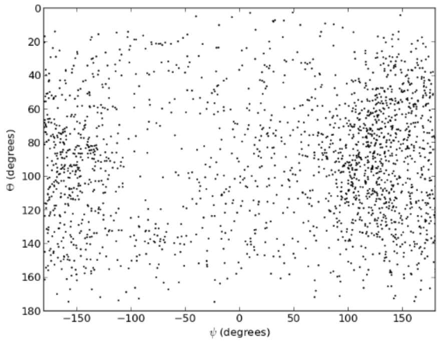

Lys-Ser-C

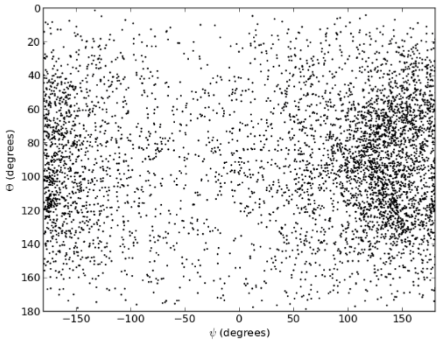

Lys-Ser-O

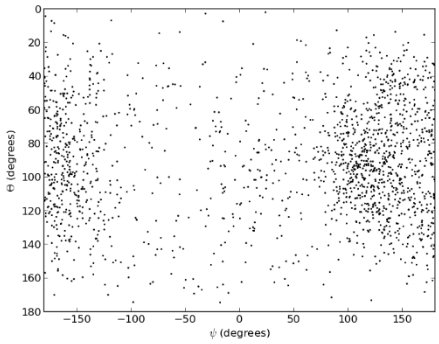

Lys-His-C

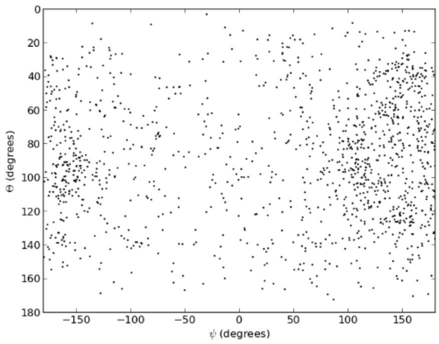

Lys-His-N

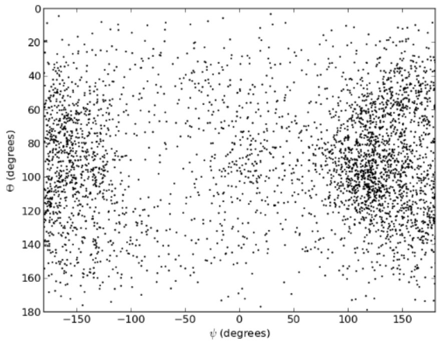

Lys-Lys-C

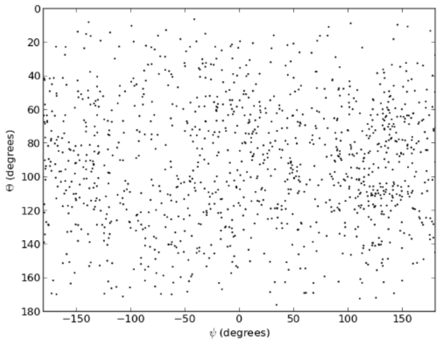

Lys-Lys-N

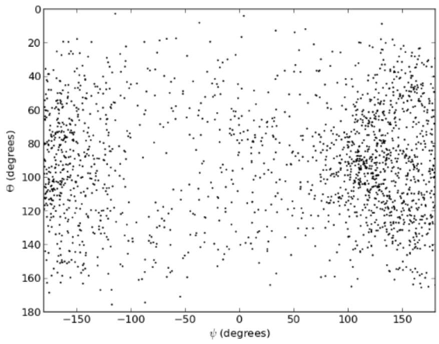

Lys-Arg-C

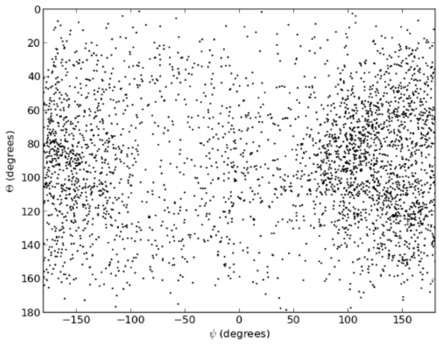

Lys-Arg-N

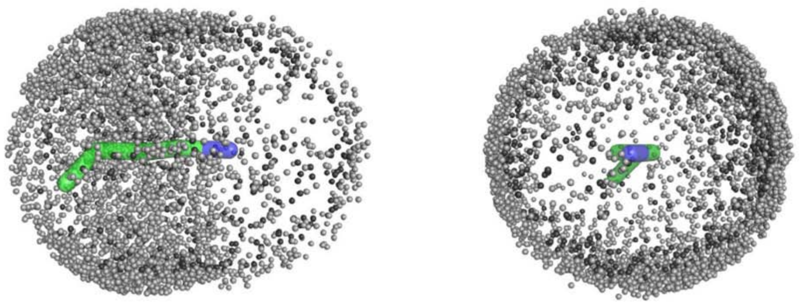

Figure S4: Distribution of Phe side chain carbon atoms around the side chain of Lys viewed from the side (left) and front (right). Dark, medium and light grey spheres represent Phe side chain carbon atoms within 3.5, 3.75 and 4.0 Å, respectively, of any of the Lys side chain Cδ, Cε or Nz atoms.

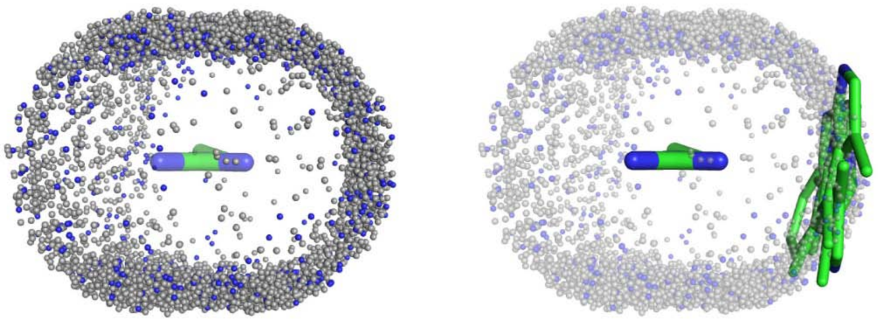

Figure S5: High in-plane density of the Trp side chain near the NH1 nitrogen of Arg in the protein data bank. The right hand panel shows a selection of structures in which the high in-plane density of Trp side chain atoms adjacent to the guanidine NH1 nitrogen represents cation-π interactions with the Trp side chain aligned so that the Arg NH1 nitrogen lies within the negative potential of the indole aromatic group.

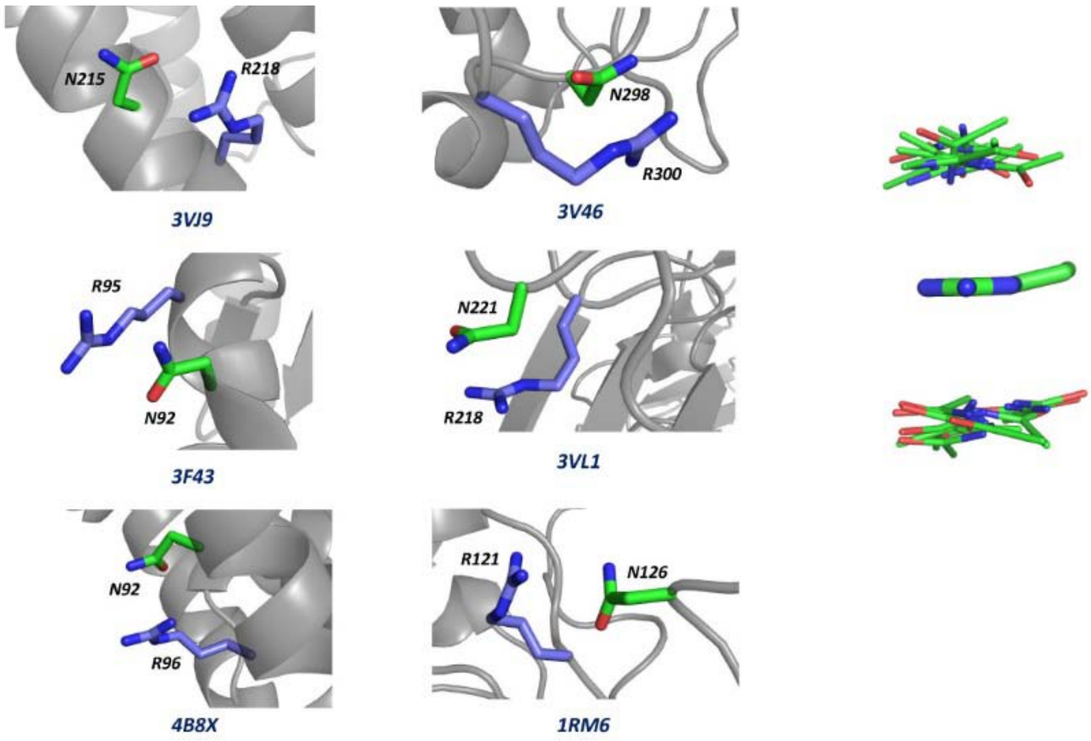

Figure S6: Arginine guanidine – asparagine side chain amide parallel stacking in protein crystal structures. The left hand series of panels shows examples from the protein databank of stacked Arg-Asn side chains in which the side chains (Arg CZ – Asn CG) are within 4.2 Å. The protein data bank accession identifier is given below each panel. A similar distance criterion applies to the overlay of multiple parallel stacked Arg-Asn side chains in the protein data bank (right panel).

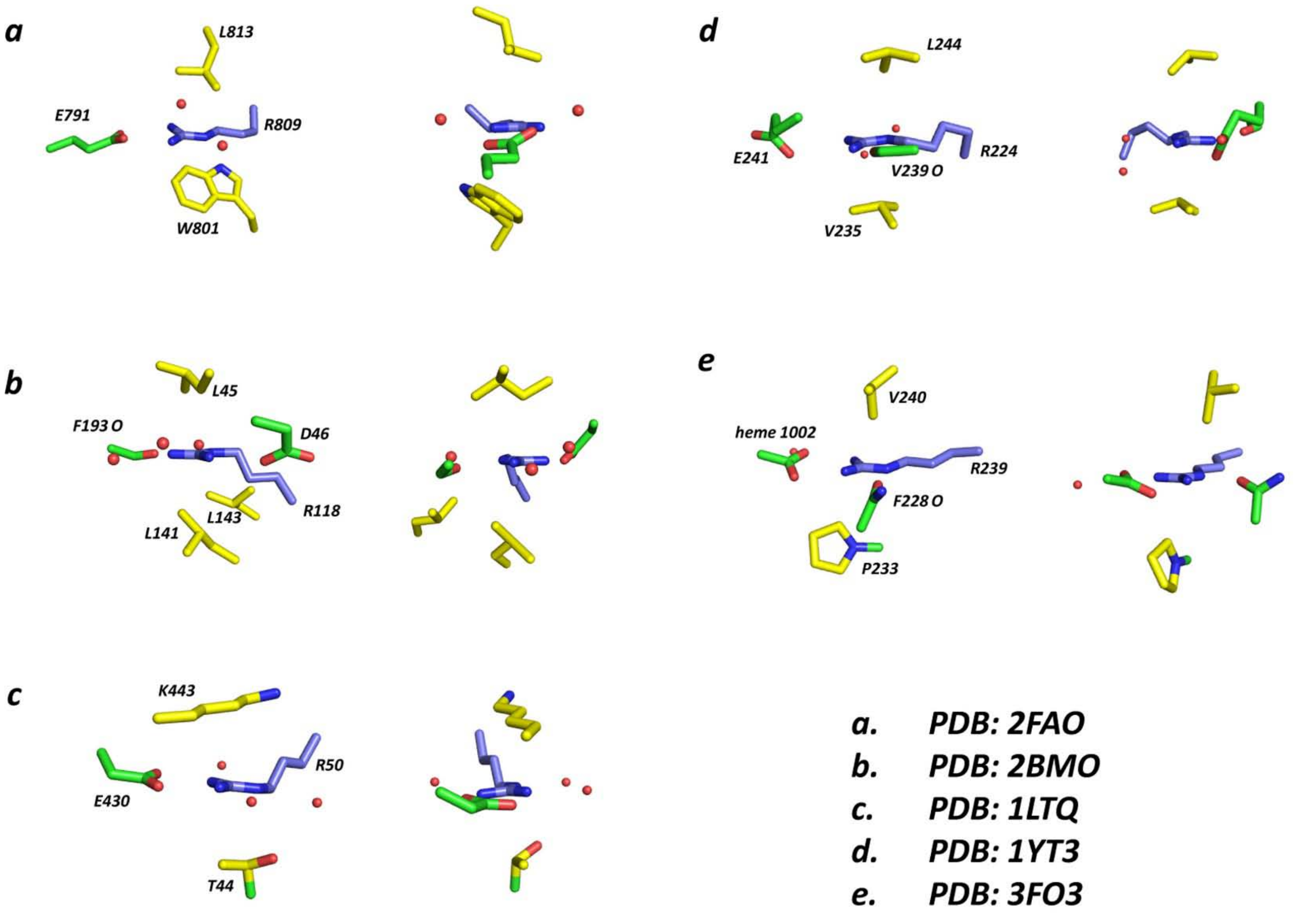

Figure S7: Selected examples of partially buried arginine side chains in protein structures in which an Arg side chain is "sandwiched" between non-polar side chains with hydrogen bonding donor oxygen atoms near in-plane positions. Non-polar side chains are yellow, polar side chains are green with carboxylate, hydroxyl, or backbone amide oxygen atoms in red. Red spheres are water oxygens. Environments of each Arg side chain (blue) are illustrated with a side (left) and front-on (right) view.

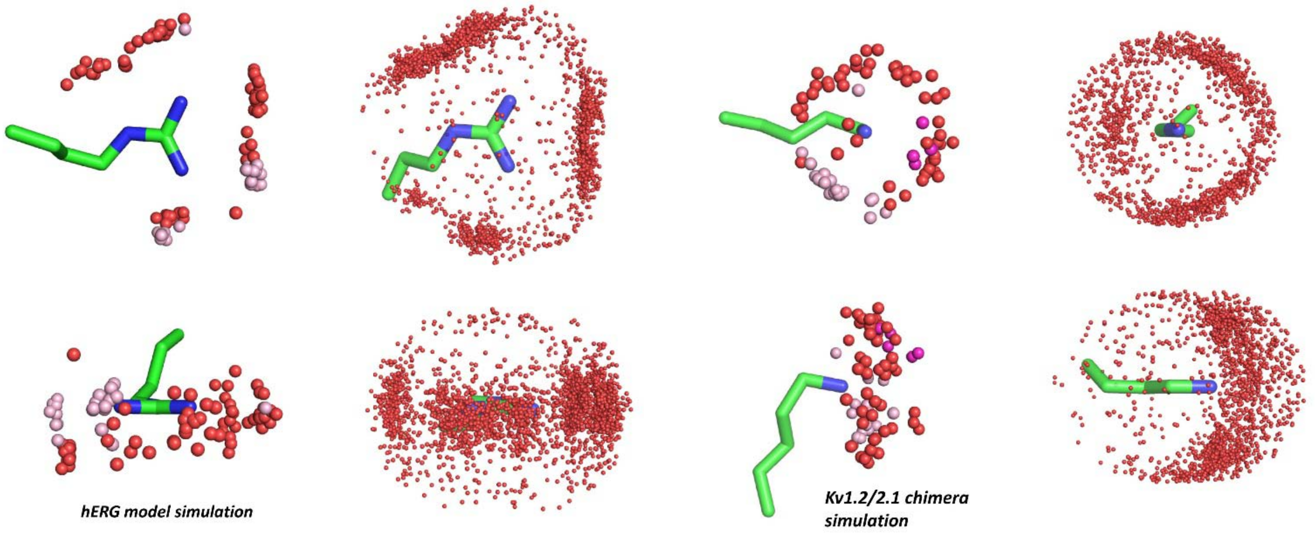

Figure S8: Comparison of side chain and water oxygen atom distributions around Arg and Lys side chains from molecular dynamics simulations of voltage sensitive ion channels, and from inspection of the protein data bank. The left hand simulation figure shows the distribution of water (red) and side chain aspartic acid (pink) oxygens within 3.5 Å of the side chain guanidine heavy atoms of R534 of a hERG model simulation, as the R534 side chain moves across the hydrophobic plug of a voltage-sensor domain in 20 ns snapshots [from Colenso et al. 2014] (top and front-on views). The right hand simulation figure shows equivalent distributions of water (red), Asp (pink) and Glu (mauve) oxygen atoms around the side chain aliphatic amino group of K302 in an MD simulation of the Kv1.2/2.1 chimera channel (Long et al., 2007) as the K302 side chain resides in the "charge transfer center" of a voltage sensor subunit [from Colenso et al. 2014] (front-on and side views). The carboxylate oxygen atom distributions from the protein data bank are reproduced from Figure 4 of the main text.

C. K. Colenso, Y. Cao, R. B. Sessions, J. C. Hancox and C. E. Dempsey (2014) Voltage sensor gating charge transfer in a hERG potassium channel model*.* *Biophys. J.* **107**, L25-L28.

S. B. Long, X. Tao, E. B. Campbell and R. MacKinnon (2007) Atomic structure of a voltage-dependent K^+ channel in a lipid membrane-like environment. *Nature* **450**, 376-382.

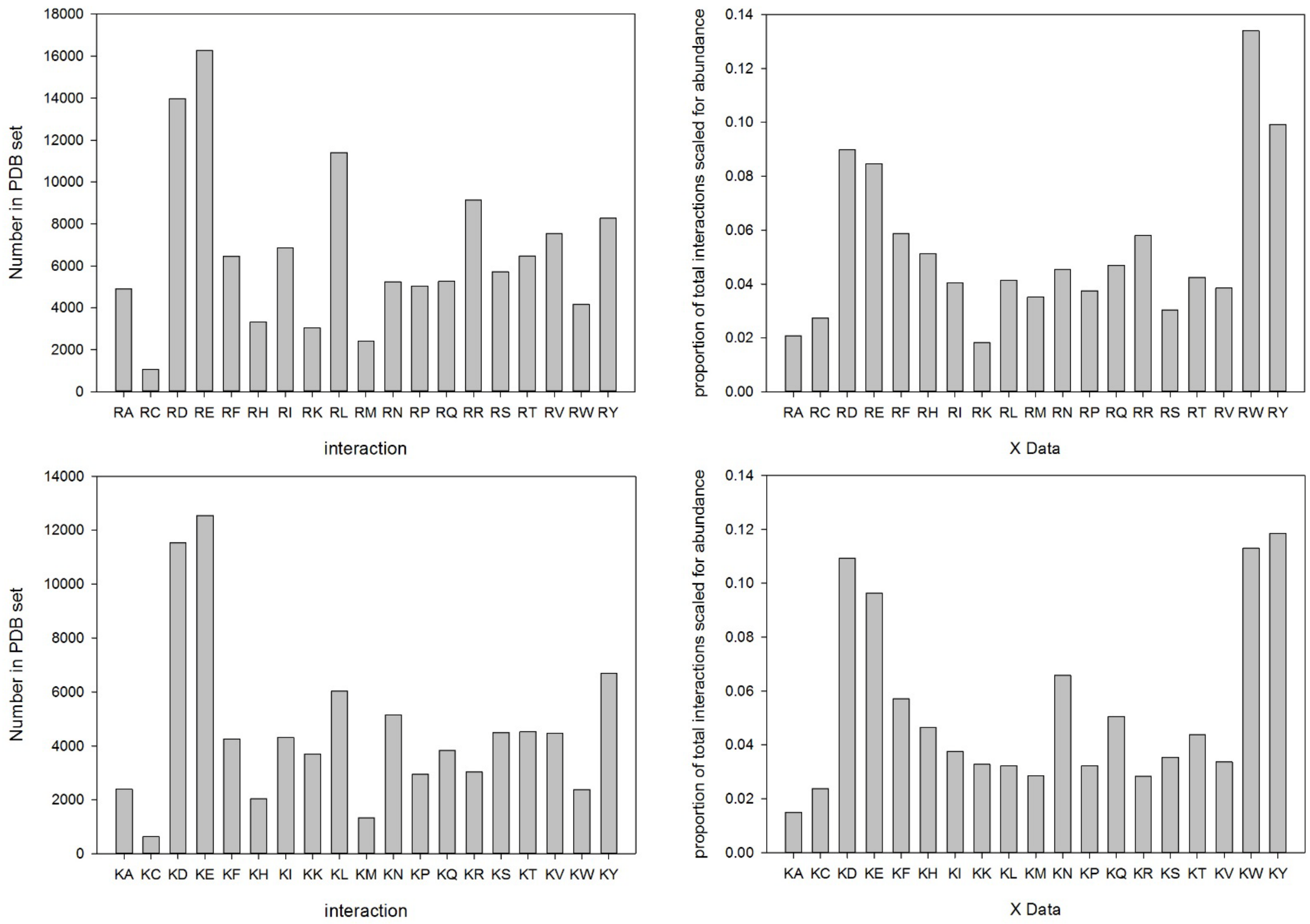

Figure S9: Numbers of “interacting” pairs of the 19 non-glycine amino acid side chains with the side chains of arginine (top) and lysine (bottom). The absolute number of interactions (amino acid side chain atoms within 5 Å of side chain atoms of Arg or Lys) is shown in the two left hand panels. Each Arg-X or Lys-X amino acid pair is counted once; i.e. if two or more atoms of a side chain are within 5 Å of Arg or Lys side chain atoms this side chain pair is counted as one interaction.

The right hand panels are scaled by dividing by the relative proportion of each amino acid in the protein data bank (see Table S2 on the following page) and normalizing with respect to a total of 1.

Table S1: Numbers of "interacting" pairs for each amino acid with the side chains of Arg and Lys as defined in the Figure S8 legend. The last column lists the percentage of each amino acid in proteins in the protein databank. The amino acid composition of the protein databank was taken from the March 4th (2015) update of the UniProtKB/Swiss-Prot protein knowledgebase release 2015_03 statistics (http://web.expasy.org/docs/relnotes/relstat.html).

around Arg	number of interactions	around Lys	number of interactions	composition in PDB %
R Ala	4886	K Ala	2392	8.26
R Val	7537	K Val	4464	6.87
R Leu	11395	K Leu	6028	9.66
R Ile	6866	K Ile	4312	5.94
R Pro	5035	K Pro	2933	4.71
R Met	2414	K Met	1332	2.41
R Cys	1071	K Cys	630	1.37
R Ser	5707	K Ser	4493	6.58
R Thr	6465	K Thr	4511	5.34
R Asn	5236	K Asn	5143	4.05
R Gln	5255	K Gln	3832	3.93
R Asp	13970	K Asp	11528	5.46
R Glu	16269	K Glu	12544	6.74
R Arg	9142	K Arg	3027	5.53
R Lys	3048	K Lys	3688	5.83
R His	3317	K His	2040	2.27
R Phe	6455	K Phe	4257	3.86
R Tyr	8265	K Tyr	6683	2.92
R Trp	4162	K Trp	2378	1.09
				(Gly 7.08)
